# The Negative Effect of Carpal Tunnel Syndrome on Sleep Quality

**DOI:** 10.1155/2014/962746

**Published:** 2014-02-17

**Authors:** Ashish Patel, Maya Deza Culbertson, Archit Patel, Jenifer Hashem, Jinny Jacob, David Edelstein, Jack Choueka

**Affiliations:** ^1^Maimonides Medical Center, Department of Orthopaedic Surgery, 927 49th Street, Brooklyn, NY 11219, USA; ^2^SUNY Downstate Medical Center, 450 Clarkson Avenue, Brooklyn, NY 11203, USA

## Abstract

*Objective*. Sleep disturbances are common in patients with carpal tunnel syndrome (CTS). This study investigates the impact of CTS on sleep quality and clarifies the magnitude of this relationship. *Methods*. This is a prospective investigation of patients with CTS. Patients responded to the Levine-Katz Carpal Tunnel and the Pittsburgh Sleep Quality Index (PSQI) questionnaires to assess symptom severity and quality, respectively. Descriptive and bivariate analyses summarized the findings and assessed the correlations between CTS severity and sleep quality parameters. *Results*. 66 patients (53F, 13M) were enrolled. Patients reported a sleep latency of 30.0 (±22.5) minutes, with a total sleep time of 5.5 (±1.8) hours nightly. Global PSQI score was 9.0 (±3.8); 80% of patients demonstrated a significant reduction in sleep quality (global PSQI score >5). Increased CTS symptom and functional severity both resulted in a significant reduction in quality and time asleep. Both significantly correlated with subjective sleep latency, sleep disturbance, use of sleep promoting medications, daytime dysfunction, and overall global PSQI score. *Conclusions*. The findings confirm the correlation of sleep disturbances to CTS, that is, significant reduction of sleep duration and a correlation to sleep quality. Patients sleep 2.5 hours less than recommended and are at risk for comorbid conditions.

## 1. Introduction

The critical relationship between sleep, health, and overall well-being is gaining greater attention. The National Sleep Foundation recommends 7 to 9 sleep hours per night for adequate rest and repair [1]. Unfortunately, sleep curtailment has become increasingly prevalent in modern day society due to higher demands, longer working hours, and the introduction of radio, television,and the internet [1]. Recent investigations have demonstrated the negative impact of declining sleep quality on the human body. It is thought that reductions in sleep quality results in longer exposure to elevated sympathetic nervous system activity and to waking physical and psychological stressors [2]. Consequently, there is growing evidence that significant correlations exist between poor sleep quality and the development of comorbid conditions including obesity, hypertension, diabetes mellitus, pain, and even death [2–9].

Patients with carpal tunnel syndrome (CTS) consistently report nighttime symptoms including multiple awakenings due to hand pain and numbness. It is thought that wrist mal-position during sleep acutely exacerbates CTS symptoms by increasing the pressure within the carpal canal [10]. For the majority of patients, night symptoms and subsequent daytime dysfunction are the impetus to seek medical intervention. While numerous reports have quantified the impact of CTS on functional limitations [10–14], the critical relationship between CTS and sleep quality is rather limited. The authors are aware of just a single report studying the effects of CTS on sleep. Using a self-reported survey in 34 CTS patients, Lehtinen et al. [15] found that CTS results in frequent nighttime awakenings, an increase in fragmented sleep, and an increase in daytime sleepiness and dysfunction versus a control database of 1600 Finns.The magnitude and correlation of the relationship between CTS symptom severity and sleep quality was not studied. Understanding this relationship will provide improved patient education regarding sleep-related symptoms and a platform for future investigations studying outcomes of CTS treatment modalities on sleep restoration.

The objective of this study was to investigate the impact of CTS on sleep quality. Specifically, this study aims to answer the following questions: (1) to what magnitude does CTS adversely affect sleep quality and duration; (2) what areas of sleep quality are affected by CTS; and finally (3) is there a relationship between CTS functional severity and the impact on sleep quality parameters as assessed by validated outcome measures.

## 2. Methods

### 2.1. Study Sample

This study is a prospective clinical investigation of consecutive patients presenting to an outpatient orthopaedic hand surgeon's clinic with symptoms consistent with carpal tunnel syndrome (CTS). Each patient was evaluated by the senior author (Jack Choueka) and enrolled into the study if they met inclusion/exclusion criteria including age over 18, history, physical examination (i.e., sensory neuropathy or pain in the hand within the median nerve distribution, thenar muscle weakness/atrophy, and positive Tinel's and Phalen's test), and EMG data consistent with CTS. Subjects were excluded if they reported the following: previous hand trauma, previous hand surgery, inflammatory arthritis, or sleep pathology (i.e., sleep apnea, restless leg syndrome, etc.). The study was approved by our institutional review board prior to patient enrollment.

### 2.2. Data Collection

Once enrolled into this study, patients were asked to provide responses to a questionnaire packet, which included basic demographic information, a visual analog scale (VAS) for sleep quality and hand pain, the Levine-Katz carpal tunnel questionnaire [16] (Boston carpal tunnel questionnaire), and the Pittsburgh Sleep Quality Index (PSQI) [17].

#### 2.2.1. Demographic Information

Demographic information collected as part of routine evaluation included age, gender, symptomatic hand, and duration of symptoms.

#### 2.2.2. Visual Analog Scale

The Visual Analog Scale (VAS) consisted of 2 separate lines: the VAS-Pain scale and VAS-Sleep scale. Patients were asked to mark the severity of hand pain with an “X” on a 100-point scale (10 cm line): “0” indicating no pain and “100” indicating the worst pain imaginable. Patients were also asked to mark their sleep quality with an “X” on a 100-point scale (10 cm line): “0” indicating poor sleep and “100” indicating excellent sleep. The measurement in millimeters was used as the VAS score.

#### 2.2.3. Levine-Katz Carpal Tunnel Questionnaire [16]

The Levine-Katz (Boston) carpal tunnel questionnaire is a frequently used patient-reported questionnaire to assess patients with carpal tunnel syndrome. There are 19 questions with 2 outcome scores: the symptom severity score (SSS) and the function status scale (FSS). The symptoms severity score includes multiple questions regarding pain and numbness/tingling and includes questions regarding sleep impact. The FSS assesses the patient's ability to perform activities of daily living. Each response is scored from 1 point (mildest) to 5 points (most severe) and averaged to obtain the SSS and FSS (scored 1–5). The Levine-Katz questionnaire has been proven to be reproducible, internally consistent, and responsive to clinical change.

#### 2.2.4. Pittsburgh Sleep Quality Index [17]

The Pittsburgh Sleep Quality Index (PSQI) is a patient-reported questionnaire which assesses sleep quality over a 1-month time interval. There are 19 questions which generate seven “component” scores (scored 0–3, 0 = no pathology, 3 = greatest pathology): C1—subjective sleep quality, C2—sleep latency, C3—sleep duration, C4—habitual sleep efficiency, C5—sleep disturbances, C6—use of sleeping medication, and C7—daytime dysfunction. The sum of the component scores yields the “global PSQI Score” (0–21). The PSQI has been shown to be internally homogenous, consistent, and clinically responsive to changes in sleep 17.

### 2.3. Statistical Analysis

Descriptive statistics including medians, standard deviations, and minimum/maximum range were used to summarize questionnaire responses where appropriate. Bivariate analysis was used to investigate associations between sleep parameters (PSQI) and CTS parameters SSS and FSS (Levine-Katz). To address known differences in sleep quality measures based on gender and age, data were also stratified by gender and age group. Three age groups were defined: “young adults” (age 25–45), “adults” (45–65), and “elderly” (65 years or older). Analyses were carried out on all subjects and then repeated on gender- and age-stratified groups. Differences between groups were examined by ANOVA. Analysis was conducted using SPSS software (Chicago, ILL) with a level of significance set at *P* < 0.05.

## 3. Results

### 3.1. Patient Sample

66 patients were enrolled into this study. There were 53 females and 13 males, with a median age of 55.4 ± 15.4 years (range = 24.9–85.7 years). 15 patients were classified as “young adults,” 27 as “adults,” and 19 as “elderly.” Age data were missing for five subjects. Approximately 33% of patients had symptoms in their right hand, 33% in their left hand, and 33% in both hands. Patients reported CTS symptoms for a median duration of 12 ± 11.1 months (range = 1–48 months). There were no significant differences in symptom duration between gender or age strata.

### 3.2. CTS Parameters

**Figure 1:**
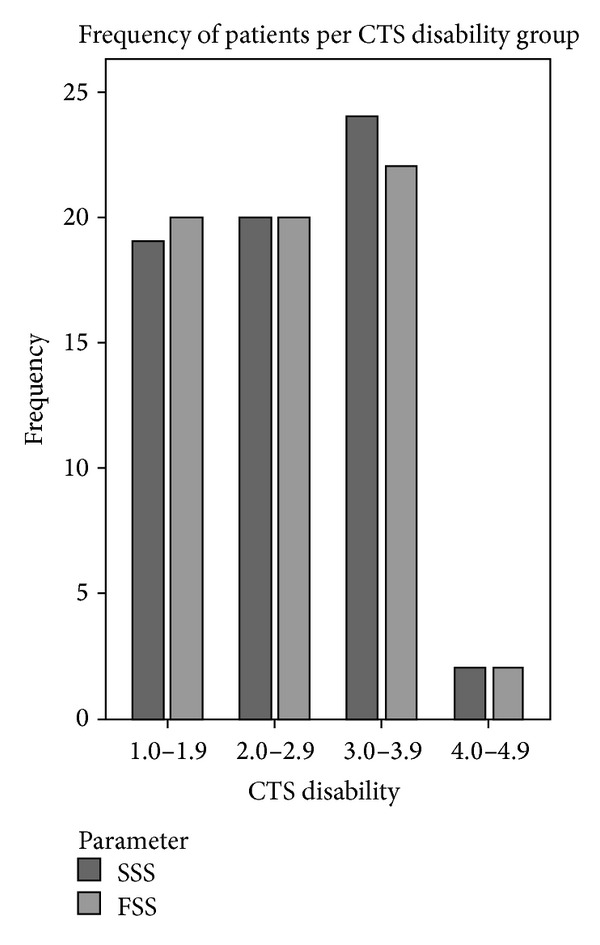
Frequency of patients versus increasing CTS disability (SSS and FSS) assessed using the Levine-Katz questionnaire.

The severity of CTS was assessed using the VAS-Pain scale and the symptom severity score (SSS) and functional status score (FSS) of the Boston carpal tunnel questionnaire. The median VAS-Pain score was 67 ± 27 mm (range = 3–100 mm). The median (standard deviation) SSS was 3.0 ± 0.8 (range = 1.5–4.5). The median FSS was 2.4 ± 0.9 (range = 1.0–4.3).  Figure 1 illustrates the distribution of patients in each CTS disability group. VAS-Pain and SSS scores showed no significant differences between genders or age groups. Females exhibited significantly higher FSS scores than males (2.8 ± 0.91 for females versus 2.0 ± 0.69 for males). There was no difference in FSS between age strata.

### 3.3. Sleep Data

Sleep quality was assessed using the VAS-Sleep scale and the PSQI questionnaire. The median (standard deviation) VAS-Sleep score was 40.5 (±28.7) mm (range = 0–100 mm). Patients reported a median (standard deviation) sleep latency of 30.0 (±22.5) minutes (range = 0–120 min) with a median (standard deviation) total sleep time of 5.5 (±1.8) hours (range = 1–10 hours). There were no significant differences between genders or age groups for VAS-Sleep scores, sleep latencies, or total sleep times.

**Figure 2:**
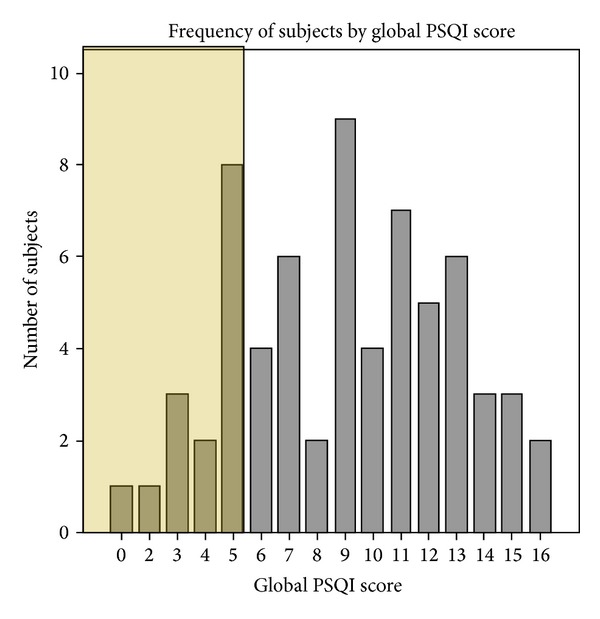
Global PSQI score over the entire study group. The shaded area is considered normal sleep.

**Figure 3:**
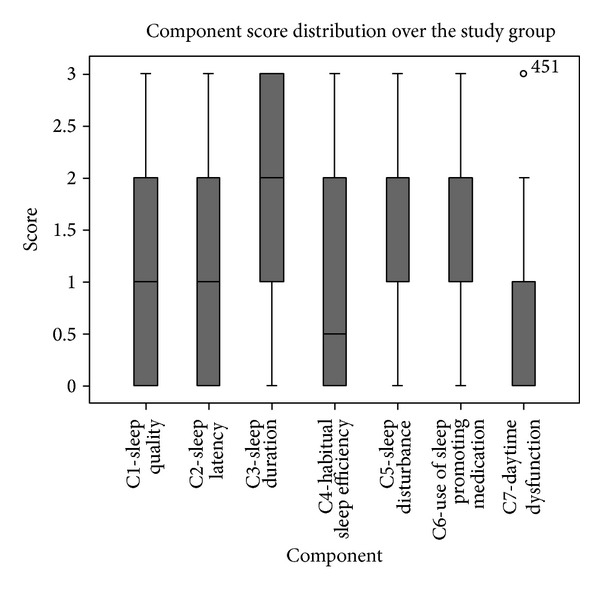
Individual sleep component analysis. Patients demonstrated sleep dysfunction in all 7 subcategories. Sleep duration, sleep disturbance, and use of sleep promoting medications were the parameters greatest effected. Bars indicate 95% min and max values.

The median (standard deviation) global PSQI score was 9.0 (±3.8) (range = 0–16). PSQI did not change significantly between genders or age groups. Global PSQI scores of greater than 5 are reported to indicate poor sleep quality [17]. 78% of patients had global PSQI scores greater than 5.  Figure 2 illustrates the distribution of PSQI score over the entire study group. Component score analysis is illustrated in Figure 3.

### 3.4. CTS and Sleep Correlations

**Figure 4:**
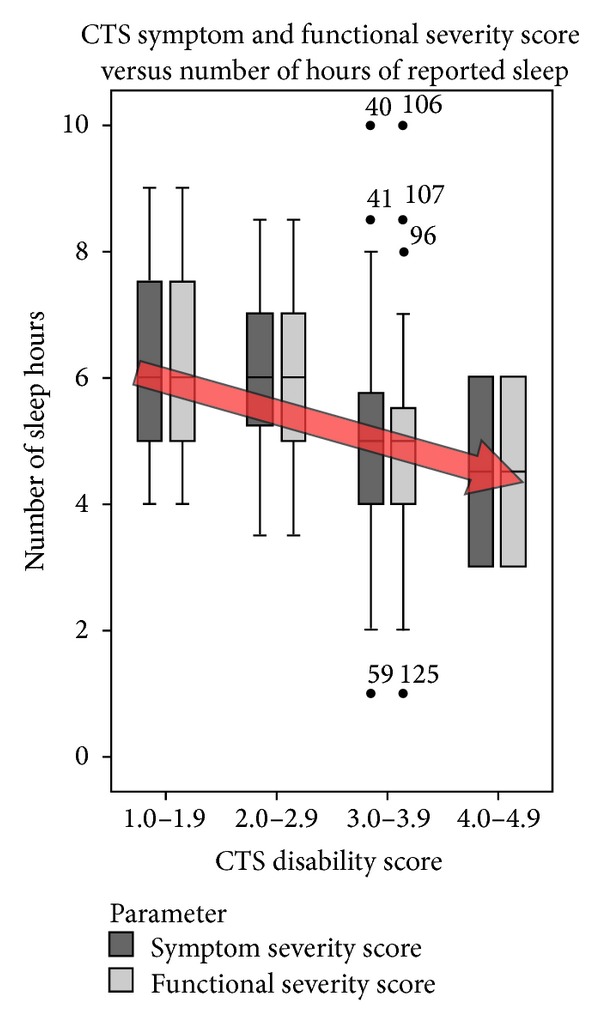
CTS disability score (SSS and FSS) versus total number of hours asleep. A significant negative correlation was demonstrated between increasing CTS and total sleep hours (*r* = −0.288, *P* < 0.023). Bars indicate 95% min and max values.

**Figure 5:**
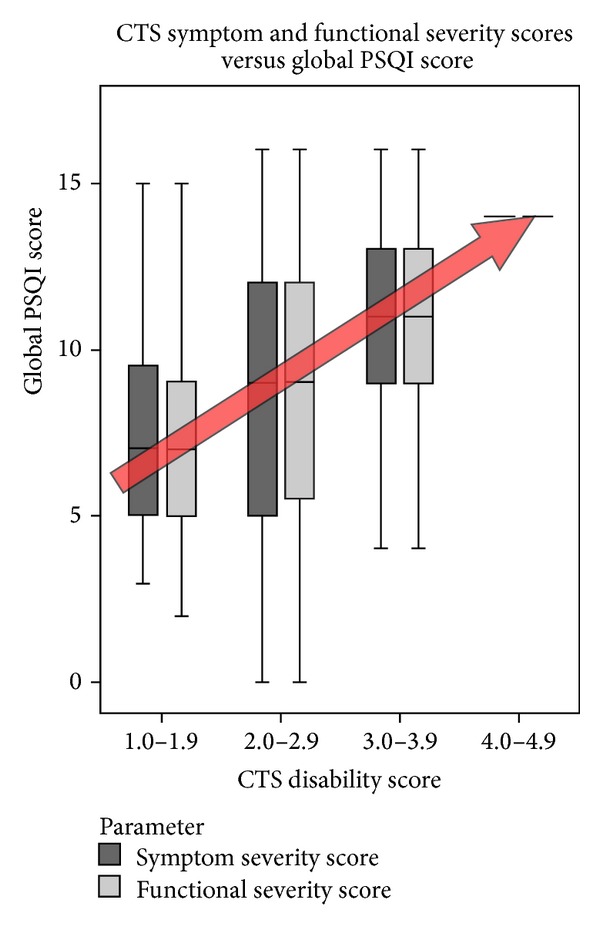
CTS disability score (SSS and FSS) versus global PSQI. A significant correlation was demonstrated between increasing CTS and increasing global PSQI score (*r* = 0.506, *P* < 0.000). Bars indicate 95% min and max values.

No correlations were evident between age, gender, or duration of symptoms and sleep parameters. Significant correlations were observed between VAS-Pain and SSS/FSS (*r* = 0.78, *P* < 0.000 and *r* = 0.57, *P* < 0.000 resp.,) and between VAS-Sleep and the PSQI (*r* = −47, *P* < 0.000). Significant correlations were evident between CTS parameters (SSS/FSS), the global PSQI score, and 5 of the 7 PSQI subcomponent sleep parameters. Increasing CTS severity (SSS and/or FSS) resulted in a significant reduction in total hours asleep (*r* = −0.288, *P* = 0.023; Figure 4) and sleep quality (*r* = −0.328, *P* = 0.008). Increasing CTS severity (SSS/FSS) also resulted in a significant increase in sleep latency (*r* = 0.339, *P* = 0.006), sleep disturbance (*r* = 0.402, *P* = 0.001), use of sleep promoting medications (*r* = 0.529, *P* < 0.000), daytime dysfunction (*r* = 0.289, *P* = 0.019), and overall global PSQI score (*r* = 0.506, *P* < 0.000; Figure 5). CTS parameters were grouped according to the symptom severity score (SSS) and functional severity score (FSS) into discrete groups (0.0–0.9, 1.0–1.9, 2.0–2.9, 3.0–3.9, and 4.0–5.0).

When subjects were examined by gender, several significant correlations were found between CTS and sleep parameters, particularly in females. Among female subjects, significant correlations were observed between VAS-Pain and VAS-Sleep as well as VAS-Pain and PSQI scores. SSS scores in females also correlated significantly with VAS-Pain, sleep latency, and PSQI. FSS scores in females correlated to VAS-Sleep scores, hours of sleep, and PSQI. Among males, the only significant correlation found was between FSS and PSQI (Table 1).

When correlations were examined in the context of age strata, several correlations were found, primarily in young adults. In subjects young adults between the ages of 25 and 45, significant correlations with the PSQI were observed in VAS-Pain as well as SSS. Likewise, FSS functional scores were correlated with VAS-Sleep scores, sleep latency, and the PSQI. Among adults aged 45–65, the only significant correlation observed was between SSS and VAS-Sleep scores. No significant correlations were observed between CTS and sleep parameters in elderly subjects aged 65 or above (Table 2).

## 4. Discussion

The effect of chronic pain syndromes (i.e., lower back pain) on sleep quality/duration is well known and has been extensively studied [18–21]. Yet CTS, the most common neurocompressive disorder, which is intimately tied to sleep symptoms, has received little attention despite the frequency of this complaint. Sleep symptoms are routinely solicited and expressed during patient presentation, although it is rarely quantified or objectively used as a parameter for treatment. The majority of attention is being directed toward the patient's pain and functional symptoms. Awareness into the deleterious effects of CTS on sleep quality can help educate patients regarding the natural course of their disease. It can also provide the physician with objective data concerning the severity of the patient's condition and potentially offer criteria for treatment.

Within our cohort, ~80% of patients demonstrated clinically significant sleep disturbances (global PSQI scores >5); a comparable group of asymptomatic subjects examined by Buysse et al., during the original characterization of the PSQI, yielded a median global score of 2.67 (±1.70) versus the median 9.0 (±3.8) observed in our cohort [17]. Furthermore, our data yielded a reported median of 5.5 hours of sleep per night, 2.5 hours less than that recommended by the National Sleep Foundation (mean 8 hours/night), and 1.2 hours less than the approximate 6.7 hours reported by control subjects in the Buysse study [1, 17]. A nightly sleep debt of this magnitude places patients at a significantly increased risk for developing or exacerbating comorbid conditions and compromising overall health and well-being.

The deleterious effects of sleep deprivation on various medical conditions have been investigated. Knutson and Turek [22] demonstrated a direct relationship between the PSQI, perceived sleep debt, and HbA1c levels; from this, the authors purport that optimizing sleep duration/quality may improve glucose regulation in type II diabetics. Gangwisch et al. demonstrated significantly increased rates of developing hypertension in subjects that slept <5 hours/night. Additionally, the 10–20% reduction in blood pressure during sleep over 24-hour periods is only partially attained by these subjects [2]. Similar findings have been confirmed by other investigators, including Sampaio et al, who found that reduced sleep quality in elderly patients correlated with higher body mass indices and increased risks of depression; this and similar studies add to the body of evidence indicating that that sleep deprivation results in significant harm to one's health [3, 4, 23–25].

Previous work on sleep disturbances in patients contending with chronic pain reinforces the notion that pain disorders result in significant sleep disruptions that can cause subsequent health problems. A study by Covarrubias-Gomez and Mendoza-Reyes on the effects of chronic, nonmalignant pain on sleep found that 89% of subjects (276/311) exhibited poor sleep quality, defined as PSQI scores of 5 or above [26]. Similarly, Shyen et al. reported increased delays in sleep onset, sleep anxiety, and sleep disordered breathing as well as night awakening episodes and parasomnias in children with idiopathic juvenile arthritis [27]. In addition to the effects of pain on sleep, recent work suggests a mechanism for the exacerbation of pain response following sleep disruption. A 2013 functional imaging study employing positron emission and magnetic resonance imaging to elucidate the relationship between pain response and mu opioid receptor (MOR) activity found a positive correlation between sleep disruption and MOR-ligand binding potential [28]. This preliminary study suggests a potential feedback loop that can further intensify both chronic pain and sleep disturbances.

During this investigation, the PSQI and the symptom severity score and functional status score from the Levine-Katz carpal tunnel questionnaire revealed important relationships between sleep and CTS. The symptom severity score, as expected, was directly correlated with the PSQI due to its inherent inclusion of sleep impact questions. More interestingly, the increasing FSS (which does not have the same direct correlation to sleep impact) demonstrated that patients experienced a linear decrease in sleep quality (global PSQI score). More specific results showed that increasing severity of CTS (SSS and FSS) was found to increase the time required to fall asleep and decreased total sleep time (PSQI questions 1 and 4). As CTS pathology worsened, so did night pain. Careful examination of questionnaire responses revealed a significant relationship between CTS severity and the frequency of night time awakenings: specifically from pain attacks (PSQI questions 6 and 13). Patients more frequently turned to sleep promoting medications (question 15) to reduce their nightly sleep debt. Additionally, escalating sleep deprivation was found to lead to increasing daytime dysfunction, possibly compromising work and social activities. This cascade of events equates to increasing morbidity to the patient and is often the impetus for seeking medical intervention.

Several limitations exist in this investigation. Firstly, although comorbid sleep pathology was queried during patient enrollment, extensive screening, that is, sleep polysomnography testing, was not implemented prior to data collection. Patients with undiagnosed sleep disturbances may have been recruited into this investigation. Secondly, additional objective measures of CTS severity (i.e., grip strength) were not collected routinely for this study. These objective data would have complemented subjective patient reported questionnaire responses. Finally, quantitative nerve conduction data was not collected to study the correlation between symptom severity, nerve injury, and sleep quality. However, Chang et al. [29] in their study from 2008 have indicated that there is no definitive correlation between the Levine Katz questionnaire (SSS/FSS) and nerve injury as assessed by electrophysiologic data. In addition, this study has raised the interesting question of a cyclic style “cause and effect” of sleep deprivation and functionality in activities of daily life where pain and dysfunction as assessed by the FSS were directly correlated to decreased sleep quality. It is likely that the decreased sleep quality further reduced the FSS score since the activities of daily living were directly affected by sleep deprivation.

CTS is the most common neurocompressive disorder in human population with approximately 250,000–300,000 carpal tunnel releases preformed in the United States each year [30]. Sleep symptoms are frequently reported in patients with CTS, although they are generally given limited attention. This study has established the preliminary relationships between subjective CTS severity (using patient-reported questionnaires) and the subsequent effect on subjective sleep quality (using patient-reported questionnaires). This study sheds some light on a critical relationship that is poorly understood and hopes to raise awareness of the negative impacts of carpal tunnel syndrome on a variety of sleep parameters. Future research in this field will attempt to investigate the impact of operative and nonoperative treatment on the resolution of CTS symptoms and the subsequent effect on sleep quality.

## Figures and Tables

**Table 1:** Significant correlations of CTS and sleep parameters by gender.

Variable	By	Correlation	*n*	*P* value
Gender: female				
VAS-Sleep	VAS-Pain	−0.3220	47	0.0273
PSQI	VAS-Pain	0.4157	47	0.0037
SSS	VAS-Sleep	−0.4358	48	0.0020
SSS	Latency	0.3391	51	0.0149
SSS	PSQI	0.5644	52	<0.0001
FSS	VAS-Sleep	−0.3498	47	0.0159
FSS	Hours of sleep	−0.2896	49	0.0435
FSS	PSQI	0.4596	51	0.0007

Gender: male				
FSS	PSQI	0.5905	13	0.0336

**Table 2:** Significant correlations of CTS and sleep parameters by age strata.

Variable	By	Correlation	*n*	*P* value
Young adults (25–45 years)				
PSQI	VAS-Pain	0.5735	14	0.0320
SSS	PSQI	0.7151	15	0.0027
FSS	VAS-Sleep	−0.6312	14	0.0155
FSS	Latency	0.7417	15	0.0016
FSS	PSQI	0.669	15	0.0064

Adults (45–65 years)				
SSS	VAS-Sleep	−0.4229	26	0.0314

Elderly (>65 years)				
No significant differences between CTS and sleep parameters				
